# *Virgibacillus halodenitrificans* ST‐1 for fermentation of shrimp paste and hydrolysates of its protease

**DOI:** 10.1002/fsn3.1777

**Published:** 2020-08-22

**Authors:** Xueqin Liu, Yanli Feng, Xiaohua Lai, Tian Deng, Xin Liu, Mingsheng Lyu, Shujun Wang

**Affiliations:** ^1^ Jiangsu Key Laboratory of Marine Bioresources and Environment /Jiangsu Key Laboratory of Marine Biotechnology Jiangsu Ocean University Lianyungang China; ^2^ Co‐Innovation Center of Jiangsu Marine Bio‐industry Technology Jiangsu Ocean University Lianyungang China; ^3^ Collaborative Innovation Center of Modern Biological Manufacturing Anhui University Hefei China

**Keywords:** antioxidant activity, enzymatic specificity, protease, salt‐tolerance, shrimp paste, *Virgibacillus halodenitrificans*

## Abstract

The nutrition and flavor of shrimp paste came from hydrolyzation by enzymes that were produced by microorganisms. The salt‐tolerant strain *Virgibacillus halodenitrificans* ST‐1 isolated from shrimp paste was studied and used in the fermentation of shrimp paste. The strain and the protease produced by ST‐1 were investigated. The optimum pH of the protease was 8.0, and the reaction temperature was 30°C. The protease showed high activity in the range of pH (5.0–11.0) and NaCl concentration (1%–15%). Divalent cations such as Ba^2+^, Ca^2+^, Mg^2+^, Mn^2+^, and Si^2+^ could enhance the protease activity. Residual activity of protease was more than 90% when it was incubated with PMSF and H_2_O_2_. Also, the enzyme retained more than 90% of initial activity after it was incubated with organic solvents. Variety of natural proteins could be substrates of the protease. By analyzing the release rate of free amino acids, it was predicted that the cleavage sites of the protease were mainly Glu, Asp, Gly, Leu, and Lys. Moreover, the hydrolysates of the protease had antioxidant activity, especially for DPPH and superoxide anion radical scavenging. The strain ST‐1 and the protease both were excellent candidates for food industries.

## Graphical abstract

Virgibacillus halodenitrificans ST‐1 was isolated from shrimp paste. The protease hydrolyzed the peptide bonds of Glu‐, Asp‐, Gly‐, Leu‐, and Lys‐. The hydrolysates were in rich of flavor amino acids and had antioxidant activity.

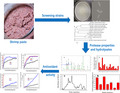

## 1 INTRODUCTION

Shrimp paste is one of the traditional foods in eastern China, even in East Asia (Phewpan et al., 2020). The fresh shrimps are fermented naturally by adding high concentration of salt. The fermentation would take over one month normally to form flavor and nutrition (Zhu et al., 2019). Proteases could hydrolysis protein effectively by breaking down the peptide bond that links amino acid to poly‐peptide chain, and they account for over half of the total world enzyme markets in the world (Amin, 2018; Moradi, Sun, Song, & Hu, 2019; Raval, Pillai, Rawal, & Singh, 2014; Uttatree & Charoenpanich, 2018). Protease from microorganisms have been studied due to their excellent characteristic and highly yield, and bacterial proteases have been exploited the most widely compared with fungi, plants, and animal (Olajuyigbe & Falade, 2014; Rekik et al., 2019).

Microorganisms secreted protease to hydrolyze the fish proteins have important functions during traditional Chinese shrimp pastes fermentation (Lv et al., 2020). Different kinds of free amino acids, peptides, oligopeptides, and ammonia were produced, and that was the key to different nutrition and flavors (Mohamed, Man, Mustafa, & Manap, 2012; Yuan, Wang, Jia, Wang, & Xu, 2017). Proteases can improve the quality and yield of shrimp oil and extend the shelf life of food (Mirzapour‐Kouhdasht & Moosavi‐Nasab, 2019; Wang et al., 2019). Although many microorganisms are known to produce protease, novel enzymes having specific properties are still required for different applications (Speranza et al., 2015; Jayakumar, Jayashree, Annapurna, & Seshadri, 2012).

Three strains of *Pediococcus* were used to ferment fish sauce under laboratory conditions (Zhang, Wang, & Mou, 2017). The fermentation method not only shortened the fermentation time, but also improved the product quality. The halophilic protease‐producing bacterium *Bacillus* isolated from shrimp paste plays a crucial role in the fermentation process. Bacterial protease contributed to the flavor formation greatly in a fermentation. Lian et al isolated a strain of *Aspergillus niger* with the ability to hydrolyze proteins from traditional fermented shrimp paste, and molds often produced good flavor in fermented foods (Lian et al., 2014). However, Lu et al. found that *Staphylococci* and *Bacillus* were the main microbial community that produced protease in shrimp paste (Lv et al., 2016). Due to a variety of harsh environments, such as osmotic pressure, pH, temperature of the naturally shrimp paste fermentation (Singh & Chhatpar, 2011; Jeong, Jung, Lee, Jin, & Jeon, 2013), the separation of protease‐producing salt‐tolerance, special flavor producing bacteria in shrimp paste was of great significance for the shrimp paste processing (Ly, Mayrhofer, & Domig, 2018).

In the traditional shrimp paste fermentation, the microbial flora works together, and the dynamics of microbial diversity happens in the processing (Phewpan et al., 2020). However, the contribution of each of bacteria did not clarified. In modern industrial production, it was important to know the role of each bacterium (Lv et al., 2020). The nutrition and flavor of shrimp paste came from proteases. The different peptides or oligopeptides were produced, and they made the food with new function (Choksawangkarn, Phiphattananukoon, Jaresitthikunchai, & Roytrakul, 2018). Additionally, proteases with novel catalytic properties and their hydrolysates could be applied in various industries such as detergent, surfactant, antibacterial substances, and oxidizing agent (Akiyama, Yamazaki, Tada, Ito, & Akiyama, 2014; Duan et al., 2014; He, Nguyen, Su, & Zhang, 2016; Hu, Ren, Zhou, & Ye, 2019; Zhang & Kim, 2010).

In this study, we selected strains which could secret protease from the shrimp paste which was very popular in local area. The growth characteristics of strain and the protease properties were investigated. Moreover, we analyzed the shrimp paste, and the hydrolysates had quantitative flavoring amino acids and antioxidant activity. The result suggested the strain and the protease were candidates for the further application in the shrimp paste producing and food industries.

## 2 MATERIALS AND METHODS

### 2.1 Sample of shrimp paste and medium

Shrimp paste and Fresh shrimp were collected from Haiwa food company located at Haizhou Bay, Lianyungang, Jiangsu Province. Samples were kept in an icebox and brought back to the laboratory within one hour for next experiments. Broth medium: beef powder 3 g/L, peptone 10 g/L, NaCl 100 g/L, distilled water, and pH 8.0; broth solid medium: beef powder 3 g/L, peptone 10 g/L, NaCl 100 g/L, Agar 20 g/L, distilled water, and pH 8.0; casein liquid medium: beef powder 3 g/L, peptone 10 g/L, casein 10 g/L, NaCl 100 g/L, distilled water, and pH 8.0; screening medium: beef powder 3 g/L, peptone 10 g/L, casein 10 g/L, NaCl 100 g/L, Agar 20 g/L, distilled water, and pH 8.0.

### 2.2 Screening and identify the strains produced protease

One gram shrimp paste was added to 20 ml of broth medium for enrichment culture at 25°C, 180 rpm for 12 hr. After slightly precipitating, 100 μl of the supernatant was spread on screening medium, and the plates were inverted in 25°C incubators. Colony which was around with a clear zone was selected and streak plate to pure the strain. Individual bacteria were isolated and stored. The strains were inoculated in casein liquid medium at 25°C and shaken at 180 rpm for 36 hr. The fermentation broth was centrifuged at 15,777 *g* for 10 min, and the supernatant was taken as a crude enzyme solution.

The purified cultured strain ST‐1 was subjected to morphological observation and physiological and biochemical identification according to the “Common Bacterial System Identification Manual” and “Berger's Bacterial Identification Manual.”

The genome of the strain was extracted using a bacterial genome extraction kit, and amplification of 16S rDNA was performed. The PCR universal primer was as follows: 27F: 5'‐AGAGTTTGATCCTGGCTCAG‐3' 1492R: 5'‐GGTTACCTTGTTACGACTT‐3', and the reaction system was as follows: PCR mix (20 μl), upstream and downstream primers (1 μl each), and DNA template 4 μl. Reaction procedure: denatured at 94°C for 5 min; denatured at 94°C for 30 s; annealing at 55°C for 30 s; extension at 72°C for 90 s, 34 cycles; final extension at 72°C for 5 min (Lai et al., 2019). The amplified products were sent to Shenggong (Shanghai China) Bioengineering Co., Ltd. for sequencing, and the resulting sequences were uploaded to GenBank. The phylogenetic tree was constructed by the Neighbor‐Joining method in MEGA7.0 software to determine the taxonomic status of the strain.

### 2.3 Growth characteristics of strain ST‐1

The seed broth of strain ST‐1 was prepared by inoculated strain into 50 ml of liquid medium incubated at 25°C, 180 rpm for 16 hr. Inoculate 2% seed liquid in broth medium, pH 8.0, rotation speed 180 rpm, liquid volume 20%, cultured at different temperatures (0–40°C) for 24 hr, Cells concentration was determined at OD600 nm. pH range 5.0–11.0, to prevent pH changes during the culture, a final concentration of 10 mM buffer was added: pH 5.0–6.0 (MES buffer), pH 6.5–7.0 (PIPES buffer), and pH 7.5–8.0 (HEPES buffer). pH 9.0–11.0 is adjusted directly with 0.1M NaOH. The medium was prepared with distilled water, the NaCl range was from 1% to 13%. Under the optimum conditions of temperature, pH, and NaCl concentration, different carbon sources were added to study the growth of the strain. The common carbon source of 5 g/L was used to replace the beef power in the basic medium. Similarly, the common nitrogen source 10 g/L common carbon source was used to replace the peptone in the basic medium, and the culture was carried out for 20 hr under the optimal conditions.

### 2.4 The properties of the protease

The protein liquid medium was inoculated by 4% of seed solution and cultured at 30°C, 180 rpm for 24 hr. After 4°C, 15,777 *g* centrifugation for 10 min, the supernatant was collected. The enzyme activity of the casein crude enzyme solution was determined at different temperatures (20–60°C) to determine the optimum temperature. The appropriate enzyme solution was taken at different temperatures for 1–5 hr, and the residual enzyme activity was determined. The enzyme activities at different pH were detected, and different pH was controlled by different buffer systems: 50 mM sodium acetate buffer (pH 4.0–6.0), 50 mM sodium phosphate buffer (pH 6.0–7.5), and 50 mM Tris‐HCl buffer (pH 7.5–9.0). The appropriate amount of enzyme solution was mixed with buffers of different pH, and the residual enzyme activities were detected after 1 hr of water bath at 25°C. The enzyme activities were measured, and the chemical reagent‐free protease was used as control. Also, the various substrates such as 2% BSA, casein, skim milk, and gelatin were used to detect the protease activity.

To investigate inhibitors of protease, PMSF, DTT; surfactant Tween 80, Triton X‐100; detergent SDS; chelators EDTA, EGTA; H2O2, metal ions (Mg2+, Fe3+, Zn2+, Ba2+, Sr2+, Hg2+, and Ca2+) and urea were chosen and mixed with protease. Different metal chloride ions and different concentrations of compounds were added, and the final concentration of metal ions was 10 mM and 50 mM, respectively. The protease was incubated with these chemicals at 25°C for 30 min, and its residual activity was measured. The protease was incubated in organic solvent such as methanol, ethanol, acetonitrile, acetone, DMSO, ether, and ethyl acetate at 25°C for 1 hr to test the stability of the protease. The residual activity was measured.

### 2.5 Protease assay

Protease activity was detected in terms of tyrosine content according to Lowry's method (Lowry, Rosebrough, Farr, & Randall, 1951). 250 μl of the protease solution was mixed with 250 μl 2% casein solution (50 mM sodium acetate buffer, pH 6.0) in 50°C water for 10 min. Then, the Folin reagent was added, and the mixed solution was detected at 680 nm. One unit of protease activity was defined as the amount of enzyme capable of hydrolyzing to 1 μg of tyrosine per min.

### 2.6 Amino acid composition of fermented shrimp paste

Fifty grams thawed shrimp was weighed, and 3% the strain ST‐1 broth was added. Then, the fermentation was carried out at 25°C for 7 days. The cultured products were centrifuged to collect the supernatant. To analyze the amino acid, 6 M HCl was added in the supernatant and hydrolyzed at 110°C for 24 hr under nitrogen. The reactive solution was cooled to room temperature and filtered. The filtered solution was mixed with ultrapure water to hold 50 ml of solution. One mL of the solution was taken and mixed with 4 ml 100 mM HCl solution to analyze the amino acid compositions using HITACHI High Speed Amino Acid Analyzer (Model L‐8900, JAPAN) at a loading amount of 20 μl.

### 2.7 Enzymatic specificity analysis

Homogenize 100 ml of shrimp paste raw material after sterilization, 5 ml of the enzyme solution was added, and the sample which was not enzymatic hydrolyzed was blank. Under the optimum temperature conditions, the solution was incubated between 1 and 24 hr under 200 rpm. After the enzymatic hydrolysis, the enzymatic hydrolysate was boiled in water for 15 min to denature the enzyme. After cooling, it was centrifuged at 7,012 *g* for 30 min. The supernatant was used to analyze the content of amino acids by an amino acid automatic analyzer (Lei, Cui, Zhao, Sun‐Waterhouse, & Zhao, 2014). Also, it was fractionated by Sephadex G‐25 gel chromatography. Each of the peaks was collected and lyophilized for antioxidant activity assays.

### 2.8 Antioxidant activity assays

The DPPH radical scavenging, the superoxide anion radical scavenging, the superoxide anion radical scavenging, and the reducing power of the hydrolysates were determined according to Wang et al. research method with a slightly modified (Zhao et al., 2017).

The scavenging effects of DPPH and superoxide anion radicals were expressed as follows:

(1) $$W\left(\%\right)=\left(1‐\frac{A_{i}‐A_{j}}{A_{0}}\right)\times 100\%$$

where *A_i_* was the absorbance of the mixture of DPPH solution and LP solution, *A_j_* was the absorbance of the mixture of LP solution and absolute ethanol, and *A*_0_ was the absorbance of the mixture of DPPH solution and water.

Where *A_i_* was sample + Tris‐HCl buffer + pyrogallol solution; *A_j_* was sample + Tris‐HCl buffer + HCl solution; and *A*_0_ was water + Tris‐HCl buffer + pyrogallol solution.

The scavenging capability of the hydroxyl radical was calculated according to following equation:

(2) $$W\left(\%\right)=\frac{A_{s}‐A_{n}}{A_{b}‐A_{n}}\times 100\%$$

where *A*_s_ was the absorbance of a sample, *A*_b_ was the absorbance of the control without a sample, and *A*_n_ was the absorbance of the reagent blank.

Determination of reducing power, in brief, 0.1 ml of LP solution was mixed with 0.1 ml of 0.2 M pH 6.6 PBS solution and 0.1 ml of 1% (w/v) K_3_Fe(CN)_6_, and the mixture was incubated at 50°C for 20 min. Then, 0.1 ml 10% trichloroacetic acid was added into the mixture. The mixture was centrifuged at 5,000 g for 10 min, and 0.05 ml of supernatant was mixed with 0.25 ml of distilled water and 0.01 ml 0.2% FeCl_3_. The mixture was then incubated at room temperature for 10 min and measured the absorption at 700 nm. All samples were in triplicate.

### 2.9 Statistical analysis

All the experiments were performed in triplicate for each sample. The data were subjected to an analysis of variance, and significance of the difference between means was determined with Duncan's multiple range test (*p* < .05) using SPSS (SPSS Statistics 20, International Business Machine).

## 3 RESULTS

### 3.1 Protease‐producing strains and identification

**FIGURE 1:**
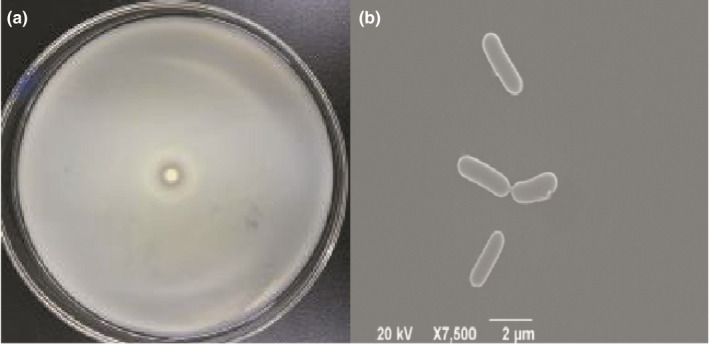
The clear zone around the colony of strainST‐1 on the plate (a) and *SEM* photograph of strain ST‐1 (×7,500) (b)

The shrimp paste liquid shaken to 16 hr was applied to casein solid agar plates, and the strains circled a transparent zone was picked. The strain numbered ST‐1 was found to produce the largest transparent zone on the casein solid plate (Figure 1a). The colony was milky white, opaque, smooth and moist, round, with neat edges, slightly protruding at the center, and easy to pick up colonies. The strain ST‐1 was observed by Gram staining, and the strain was Gram‐positive. The colony was small, round, low convex, microtransparent to opaque, and the cells were rod‐shaped (Figure 1b).

**FIGURE 2:**
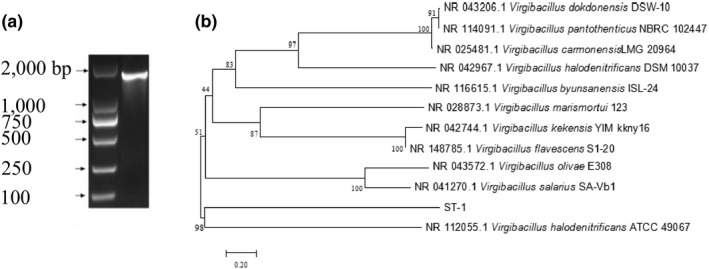
The 16S rDNA PCR products in agarose (a) and the phylogenetic tree of strain ST‐1 (b)

The 16S rDNA PCR products were sequenced to obtain a sequence of 1529 bp. After sequence alignment, the similarity between strain ST‐1 and *Virgibacillus halodenitrificans* was found to be 98.9%, and the phylogenetic tree is shown in Figure 2.

ST‐1 and *V. halodenitrificans* have highly similarities in physiological and biochemical characteristics (Table 1). It was different on the branch characteristics, such as oxidase and decarboxylase. Combined with results of the physiological, biochemical characteristics, and molecular identification, the ST‐1 strain is *V. halodenitrificans*.

**TABLE 1:** Differences between strain ST‐1 and *Virgibacillus halodenitrificans*

Characters	ST‐1	*V. halodenitrificans*
G^+^/G^‐^	+	V
0°C	−	−
Temperature (°C)	10–45	10–45
NaCl (%)	1–15	2–23
pH	6.0–10.0	5.8–9.6
Casein	+	+
Gelatin	+	+
Urea	−	−
Methyl red	−	−
Oxidase	−	+
V‐P	−	−
Arabinose	−	−
H_2_S	−	−

Note

1. Strain ST‐1; 2. *V. halodenitrificans*.

### 3.2 Growth characteristics of strain ST‐1

**FIGURE 3:**
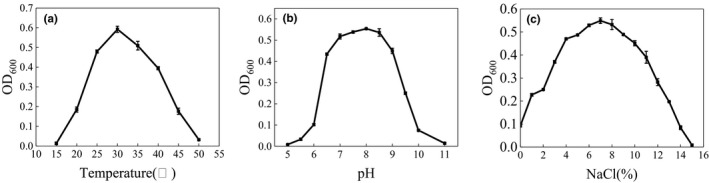
The strain growth was affected by temperature (a), pH (b), and NaCl concentration (c)

The strain ST‐1 was growth well at the temperature between 25°C and 30°C. With increasing temperature, the growth of ST‐1 decreased sharply (Figure 3a). As shown in Figure 3b, the optimum growth pH of the strain ST‐1 was 8.0. The results showed the strain ST‐1 was very sensitive to the change of pH. The strain ST‐1 could tolerant higher concentration of sodium chloride. The optimum growth was in 7% NaCl medium. When the concentration of sodium chloride was higher than 9%, the growth ability of the strain decreased significantly (Figure 3c).

**FIGURE 4:**
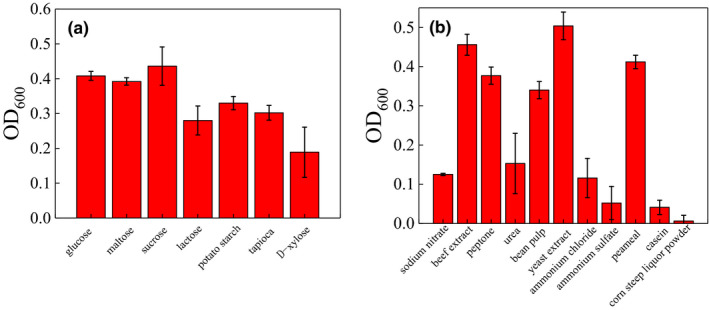
Effect of (a)carbon source and (b) nitrogen source on the growth of strain ST‐1

The growth of the strain was measured after cultured with different carbon source, and the sucrose could promote the growth significantly (Figure 4a). For the nitrogen source, the yeast extract and beef extract could enhance the growth of the strain ST‐1significantly (Figure 4b). The results showed the strain ST‐1 preferred to grow in the medium that contained more protein (Table 1).

### 3.3 Characteristics of the protease

**FIGURE 5:**
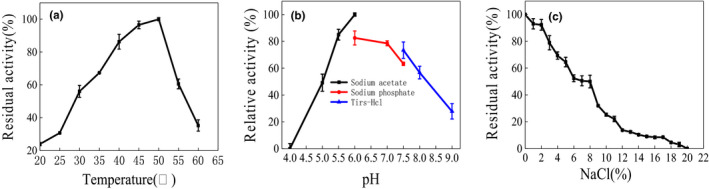
The effect of temperature(a), pH(b), and NaCl concentration (c)

The optimum temperature of protease activity was 50°C. It was sensitive to higher temperature, but the activity combined with temperature from 25 to 50°C, as linear (Figure 5a). pH 6.0 was optimum condition for the protease. The enzyme activity remained above 80% between pH 5.5 and pH 7.5 (Figure 5b). The protease activity decreases with the increase of NaCl concentration (Figure 5c). The protease could keep 50% activity in 8% NaCl condition. The results showed activity can remain above 80% in the concentration range of 4% NaCl.

The effects of 10 and 50 mM metal ions on the protease activity are shown in Table 2. Ca^2+^, Mg^2+^, K^+^, Ba^2+^, Co^2+^, Mn^2+^, Si^2+^, and Na^2+^ could promote the activity; on the contrast, Cu^2+^, Fe^3+^, Zn^2+^, Li^+^, and Cd^2+^ could inhibit the enzyme activity to a large extent. The Cu^2+^ could denature the protease on the both concentrations specially.

**TABLE 2:** Effect of metal ions on the activity of protease

Reagents	Residual activity (%) (10 mM)	Residual activity (%) (50 mM)
Control	100 ± 1.714	100 ± 1.666
Ba^2+^	130.182 ± 0.333	163.4094 ± 0.214
Ca^2+^	140.888 ± 1.856	195.333 ± 0.0.762
Mg^2+^	141.458 ± 0.1	163.409 ± 0.214
K^+^	134.055 ± 7.188	157.87 ± 4.403
Cu^2+^	0	0
Fe^3+^	7.973 ± 0.476	6.122 ± 0.999
Zn^2+^	29.954 ± 0.999	1.312 ± 1.642
Li^+^	129.043 ± 0.143	85.422 ± 0.524
Cd^2+^	37.358 ± 1.333	1.166 ± 0.619
Co^2+^	43.622 ± 3.380	112.827 ± 2.71334
Mn^2+^	123.121 ± 1.190	142.418 ± 2.166
Si^2+^	135.535 ± 3.999	191.106 ± 0.167
Na^+^	117.54 ± 8.759	176.529 ± 2.214

Note

Residual activity represents of mean ± *SD* of triplicates

The protease could tolerant organic solvent such as Tween 80, methanol, PMST, DMSO, acetonitrile, and acetone, and the residual activities were 113%, 97%, 96%, 95%, 89%, and 80%. However, some of organic solvent cold affect the activities that shown in Table 3. When the EDTA and EGTA were mixed with protease, the activity was inhibited totally. It may suggest the protease was a metal enzyme.

**TABLE 3:** Effect of organic solvent on the activity of protease

Solvents	Residual activity (%)
Control	100 ± 3.427
Ethanol	66.0 ± 7.045
Ethyl acetate	41.0 ± 1.428
Acetonitrile	89.0 ± 3.71
Methanol	97.0 ± 5.33
Acetone	80.0 ± 1.904
Dimethyl sulphoxide	95.0 ± 7.61
Diethyl ether	54.0 ± 0.286
PMST	96 ± 1.24
DTT	6 ± 4.0
Triton X−100	52 ± 0.38
Tween 80	113 ± 3.90
SDS	56 ± 17.23
EDTA	0
EGTA	1 ± 0.85

Note

Residual activity represents of mean ± *SD* of triplicates

The highest activity was performed when the substrate was casein. Also, the protease could hydrolyze different substrates such as power of skim milk, gelatin, BSA, azocasein, and hemoglobin (Table 4).

**TABLE 4:** Substrate specificity of *protease*

Natural proteinaceous substrates	Residual activity (%)
Casein	100 ± 7.14
skim milk	64.71 ± 3.57
BAS	26.83 ± 0.10
gelatin	33.34 ± 0.57
Hemoglobin	8.08 ± 0.29
Azocasein	15.23 ± 2.57

Note

Residual activity represents of mean ± *SD* of triplicate

### 3.4 Amino acid composition of fermentation product

**FIGURE 6:**
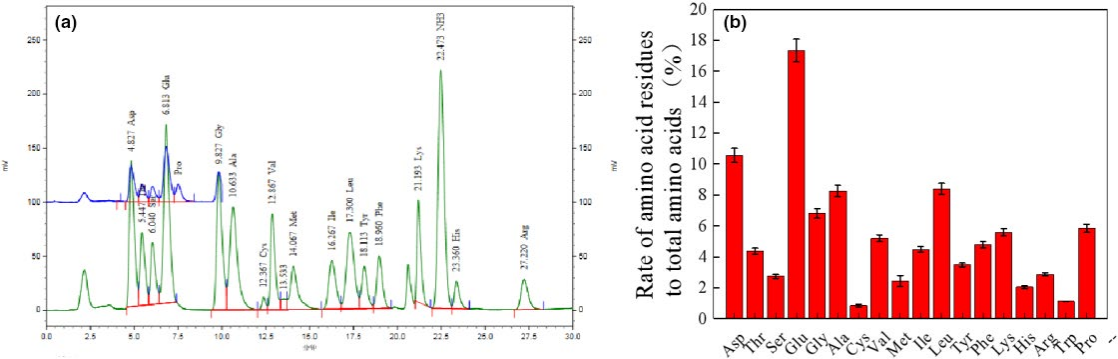
Amino acid composition of fermentation product, (a) reported by HITACHI High Speed Amino Acid Analyzer, (b) analyzed the composition

The amino acid of fermentation products was analyzed (Figure 6a), and the composition of amino acid is shown in Figure 6b. After 7 days of fermentation, the protease could release different kind of amino acid. As showed in the figure, glutamic acid was more than the others significantly.

### 3.5 Enzymatic specificity of the protease

The release rate of the amino acid could be used to predict the cleavage site under certain conditions. In the experiment, the free amino acid content had a good linear relationship when the shrimp was hydrolyzed by the protease. The release rate is shown in Table 5. The fastest release rates are Glu, Asp, Gly, Leu, and Lys. It was suggested that the protease produced by *V. halodenitrificans* ST‐1 preferentially hydrolyzed the peptide bonds of Glu‐, Asp‐, Gly‐, Leu‐, and Lys‐ (Figure 6).

**TABLE 5:** The rate of release of AAs from shrimp hydrolyzed by the protease

AA	*V*	*R* ^2^	AA	*V*	*R* ^2^
Asp	7.13 × 10^–2^	0.9573	Ile	3.22 × 10^–2^	0.9921
Thr	2.36 × 10^–2^	0.9858	Leu	5.37 × 10^–2^	0.943
Ser	1.72 × 10^–2^	0.9893	Tyr	2.74 × 10^–2^	0.9349
Glu	7.52 × 10^–2^	0.9337	Phe	3.56 × 10^–2^	0.9971
Gly	6.90 × 10^–2^	0.9816	Lys	4.70 × 10^–2^	0.9706
Ala	3.96 × 10^–2^	0.9312	His	1.47 × 10^–2^	0.9192
Cys	9.60 × 10^–3^	0.9948	Arg	3.70 × 10^–2^	0.9961
Val	3.66 × 10^–2^	0.995	Trp	2.26 × 10^–2^	0.9987
Met	2.88 × 10^–2^	0.9897			

Note

*V* and R represented the slope and correlation of the linear relationship between free amino acid content and hydrolysis time, respectively.

### 3.6 Purification of hydrolysates and antioxidant activity

**FIGURE 7:**
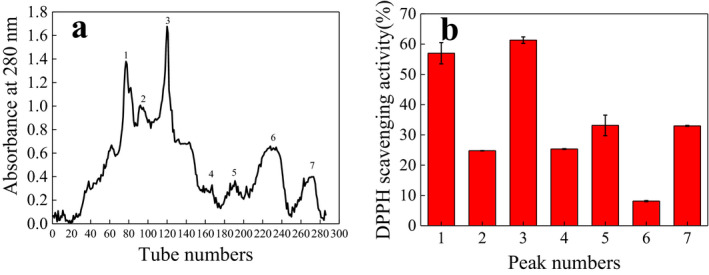
(a) The elution curve of hydrolysates by Sephadex G‐25 and (b) the DPPH radical scavenging by different peak at 1.0 mg/ml

**FIGURE 8:**
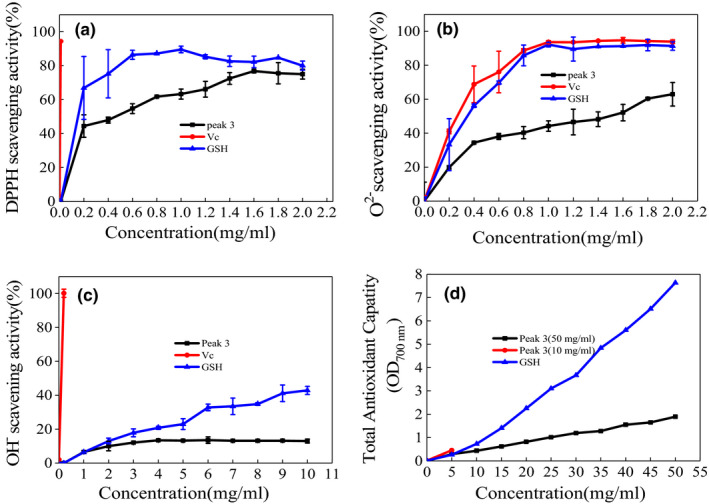
Antioxidant activities of the peak 3 of the hydrolysates. (a) DPPH radical scavenging, (b) superoxide anion radical scavenging, (c) hydroxyl radical scavenging, and (d) determination of reducing power

0.5 mg/ml of peak 3 (Figure 7) could reach 50% of DPPH radical scavenging (Figure 8a); 1.5 mg/ml of peak 3 could reach 50% of superoxide anion radical scavenging (Figure 8b). Also, the higher increase the concentration of peak 3, the stronger reducing power (Figure 8d).

## 4 DISCUSSION

After screening and identification the morphological, physiological, and biochemical characteristics, phylogenetic analysis (Ghauri, Khalid, Grant, Grant, & Heaphy, 2006), we fund the highest copy strains and highest protease activity was *V. halodenitrificans*. So, we decided to study it deeply. *V. halodenitrificans* RSK CAS1 was performed to determine the optimal medium and optimal culture conditions. Its optimum NaCl concentration is 15.32 g/L (Sathishkumar, Ananthan, & Raghunathan, 2015). *V. halodenitrificans* SK1‐3‐7 isolated from the fish sauce could produce protease which could tolerant 0.5 M NaCl (Montriwong, Rodtong, & Yongsawatdigul, 2015). *V. halodenitrificans* ST‐1 could grow in a halobiotic condition, and the optimal NaCl concentration was 70 g/L. Meanwhile, the protease of *V. halodenitrificans* ST‐1 can maintain the enzyme activity above 80% in the concentration range of 0%–3% NaCl. Compared with *Virgibacillus* sp. P‐4, it can shorten the fermentation time of shrimp paste (Zhang et al., 2017). The strain ST‐1 which could grow well in 1%–15% NaCl was more suitable for fermentation of marine products.

The protease of *V. halodenitrificans* ST‐1 could not be affected high‐concentration divalent metal ions (Ba^2+^, Ca^2+^, Mg^2+^, Co^2+^, Mn^2+^, Si^2+^, and Na^2+^) and almost unaffected by some organic solvents such as Methanol and Dimethyl sulphoxide. The characteristic makes the protease expand its application (Fang et al., 2009).

The cleavage site was predicted by analyzing the release rate of free amino acids in the enzymatic hydrolysate. Yanjie Zhang et al. studied the cleavage sites of *Virgibacillus* sp. P‐4 as Phe‐, Tyr‐, Lys‐, His‐, Pro‐, and Leu‐ (Zhang et al., 2017). The protease produced by *V. halodenitrificans* SK1‐3‐7 preferably hydrolyzed Suc‐Ala‐Ala‐Pro‐Phe‐pNA (Montriwong et al., 2015). Our research found the cleavage sites of the protease of *V. halodenitrificans* ST‐1 were Glu‐, Asp‐, Gly‐, Leu‐, and Lys‐. The quantity of glutamic acid, umami amino acid, was released significantly higher, and the flavor will be enhanced.

The DPPH scavenging and superoxide anion radical scavenging were higher than the products from marine animal *Tergillarca granosa* (Ganesan et al., 2020), *Eu* *polyphaga sinensis* walker (Zhang et al., 2019). The antioxidant activity came from peptides or oligopeptide of hydrolysates (Hu et al., 2019; Liang, Wang, Li, Chu, & Sun, 2019). After fermentation of *V. halodenitrificans* ST‐1, the shrimp paste was not only rich in flavoring amino acids, but alto rich in peptides with antioxidant activity that could improve immune system of the consumers (Kleekayai et al., 2015). From the perspective of food seasoning production, the both strain ST‐1 and protease have a high application prospect.

## 5 CONCLUSION

*Virgibacillus halodenitrificans* ST‐1 was isolated from shrimp paste of Haizhou Bay, Lianyungang. The characteristic of protease produced by ST‐1 was studied. The optimal activity pH and temperature were 8.0 and 30°C, respectively. The protease was stable at a wide range of pH (3.0–11.0) and temperature (15–45°C). More than 90% residual activity was observed when the enzyme was incubated with determined organic solutions. The protease activity could be enhanced by divalent cations such as Ba^2+^, Ca^2+^, Mg^2+^, K^+^, Mn^2+^, Si^2+^, and Na^+^ and inhibited by Cu^2+^, Fe^2+^, and Zn^2+^. The protease could hydrolyze various native proteinaceous substrates such as BSA, casein, skim milk, gelatine, azocasein, and hemoglobin. The protease preferentially hydrolyzed the peptide bonds of Glu‐, Asp‐, Gly‐, Leu‐, and Lys. The hydrolysates of the protease had antioxidant activity, especially for DPPH and superoxide anion radical scavenging. The strain ST‐1 and the protease both were have a high application prospect in food industry.

## CONFLICT OF INTEREST

The authors confirm that there is no conflict of interests regarding this paper.

## ETHICAL APPROVAL

This article does not contain any studies with animals performed by any of the authors.
